# Quelle est la place de la toxine botulique dans le traitement de l’hyperplasie bénigne de prostate?

**DOI:** 10.11604/pamj.2016.24.309.4731

**Published:** 2016-08-11

**Authors:** Adil Mazdar, Ali Bedouche, Sinane Zoughari, Ahmed Ibrahimi, Hachem El Sayegh, Ali Iken, Lounis Benslimane, Yassine Nouini

**Affiliations:** 1 Service d’Urologie A, CHU Ibn Sina, Rabat, Maroc

**Keywords:** Prostate, hyperplasie bénigne de la prostate, toxine botulique, traitement médical, Prostate, benign prostatic hyperplasia, Bbtulinum toxin, medical treatment

## Abstract

La toxine botulique (TB) a fait son apparition en urologie dans le champ de la neuro-urologie comme traitement de la dyssynergie vésico-sphinctérienne et de l'incontinence par hyperactivité détrusorienne neurogène. Son action est maintenant clairement démontrée et constitue désormais un traitement largement utilisé dans l'hyperactivité vésicale neurogène. La toxine botulique peut-elle constituer un traitement efficace de l'hyperplasie bénigne de prostate (HBP)? L’injection intraprostatique de TB a récemment été étudiée dans le traitement de l’HBP. Elle semble permettre une amélioration importante des symptômes urinaires sans effets secondaires significatifs et pourrait donc représenter une alternative mini-invasive intéressante aux traitements classiques de l’HBP. Les avantages de son utilisation sont la simplicité de sa mise en oeuvre, l'absence d'effets secondaires rapportés et sa durée d'action prolongée. Des études soigneusement menées, prospectives, contrôlées portant sur des effectifs plus grands sont désormais nécessaires pour confirmer ces premiers résultats. Nous proposons a à travers ce travail une exposition des connaissances actuelles des mécanismes d’action de la TB au niveau prostatique et les résultats des principales études cliniques dans l’HBP.

## Abstract (en)

Botulinum toxin (BT) was first used in urology for the treatment of neuro-urological disorders such as bladder sphincter dyssynergia and incontinence due to neuropathic detrusor overactivity. Its action is now clearly demonstrated and it is now widely used in treating neurogenic neuropathic detrusor overactivity. May the botulinic toxin constitute an effective treatment for benign prostatic hyperplasia (BPH)? Intraprostatic BT injection has recently been investigated as a treatment for BPH. It appears that intraprostatic BT injection produced a statistically significant improvement in urinary symptoms with no significant side effects and may therefore represent an interesting minimally invasive alternative to conventional treatment of BPH. The benefits of using it are the simplicity of its implementation, the lack of reported side effects and a longer duration of action. Carefully conducted prospective, controlled studies on larger number of cases are now needed to confirm these early results. This study aims to assess the state of current knowledge of the mechanisms of action of BT in prostate disease and the results of major clinical trials for BPH.

## Introduction

La toxine botulique a fait son apparition en urologie dans le champ de la neuro urologie comme traitement de la dyssynergie vésicosphinctérienne et de l'incontinence par hyperactivité détrusorienne neurogène [1]. Son action est maintenant clairement démontrée et constitue désormais un traitement largement utilisé dans l'hyperactivité vésicale neurogène depuis les premiers travaux de SCHURCH [2]. Elle agit en bloquant la jonction neuromusculaire et empêche ainsi les contractions des fibres musculaires lisses vésicales. L'une des composantes de l'HBP est également une augmentation du nombre et de la contractilité des cellules musculaires lisses prostatiques. Plusieurs équipes ont donc tenté d'agir sur cette composante en réalisant des injections intraprostatiques de TB [3]. La TB est une neurotoxine produite par la bactérie clostridium botulinum. Elle a pour particularité de pouvoir réaliser un blocage neuromusculaire pré synaptique spécifique, localisé, temporaire mais de longue durée. Différentes souches de clostridium botulinum peuvent produire sept sérotypes distincts de toxine botulique. Cinq ont une activité pharmacologique chez l'homme (A, B, E, F, G), les autres sont inactives. Seules deux sont utilisées en thérapeutique, le type A (TB-A) est le plus répandu et le plus puissant, le sérotype B est le plus récent et a une durée d'action plus courte [4].

D'une manière générale, la TB-A agit au niveau de la jonction neuromusculaire en bloquant la libération d'acétylcholine (ACh) par inhibition de l'ACh estérase au niveau du neurone présynaptique. Les effets postsynaptiques sont indirects et secondaires au défaut de libération du neuromédiateur (ACh). Ce blocage entraîne une dégénérescence des transmissions nerveuses et une paralysie dans le territoire concerné. Cette paralysie est temporaire car les transmissions nerveuses se reconstituent dès que la toxine est épuisée [5]. En urologie, la toxine botulique est utilisée depuis plusieurs années dans le traitement de l'hyperactivité détrusorienne résistant aux traitements médicaux [6]. Une posologie de 200 à 300 UI est habituellement recommandée. Cette posologie est largement supérieure à celle utilisée en médecine esthétique mais à notre connaissance, aucune manifestation systémique grave n'a été rapportée [5].

## Méthodes

La recherche bibliographique a été effectuée grâce à la base de données PubMed^®^ en utilisant les mots-clés anglais suivant: « benign prostatic hyperplasia», « botulinum toxin » et « Medical treatment » jusqu'en Décembre 2013. Les publications du plus haut niveau de preuve ont été sélectionnées.

### Etat actuel des connaissances

#### Du poison à l'agent therapeutique ubiquitaire

Il a fallu attendre plus de 50 ans après la découverte du Clostridium Botulinum par Pierre Emile VAN ERMENGEM en 1895, puis l'isolement et la purification de la TB (de type A) par Hermann SOMMER au début des années 50, pour qu'un usage thérapeutique potentiel des TBs soit envisagé [7]. C'est Vernon BROOKS qui rapporta le premier qu'une injection intramusculaire directe de TB-A dans un muscle hyperactif ex-vivo y induisait une relaxation réversible [8]. En 1973 Alan SCOTT, ophtalmologue, rapportait l'effet d'une injection de Toxine botulique A dans un muscle oculomoteur chez le singe [9] en 1980 il publiait la première application humaine dans le traitement d'un strabisme [10]. Ainsi depuis le début des années 80 les TBs (type A et B) sont couramment utilisées en clinique humaine, sous forme d'injections focales intra-tissulaires.

L'usage de la TB-A en urologie fut rapporté pour la première fois par DYSKTRA en 1988, qui l'injecta dans le sphincter strié urétral pour traiter la dyssynergie vésico-sphinctérienne chez des hommes blessés médullaires [1]. SCHURCH en 2000, décrivit l'effet des injections intradétrusoriennes sur l'incontinence par hyperactivité détrusorienne neurogène [2]. RAPP en 2003 utilisa la même approche que Schurch pour traiter le syndrome clinique d'hyperactivité vésicale chez des patients non neurologiques [11]. MARIA la même année rapporte l'effet des premières injections de TB dans le parenchyme prostatique pour traiter les manifestations clinique de l'adénome [12]. Actuellement quatre spécialités pharmaceutiques de TB sont disponibles sur le marché Français: Botox^®^ et Vistabel^®^ (TBA, Allergan, vistabel étant réservé à l'usage cosmétique), Dysport^®^ (TB-A, Ipsen) et Neurobloc^®^ (TB-B, Elan).

#### Voies d'abord de l'injection prostatique

Différentes voies d'abord ont été testées pour l'injection intraprostatique de TB-A. La voie transpérinéale a été utilisée au départ sous contrôle échographique transrectal. Puis la voie transurétrale [11] a été préférée, avec comme principal inconvénient de nécessiter la réalisation d'une cystoscopie et d'une anesthésie générale. Cette technique, très proche de celle des biopsies prostatiques, semble être la voie d'abord la plus simple car elle ne nécessite ni anesthésie, ni hospitalisation. De plus, elle est bien connue des urologues qui l'utilisent quotidiennement pour la réalisation de biopsies prostatiques.

## Discussion

Les premiers résultats publiés sur l'utilisation de la toxine botulique en injections intra prostatiques dans l'HBP sont très encourageants, à notre connaissance, dix études cliniques ont été publiées par huit équipes différentes. Toutes les études ont mis en évidence une amélioration significative du score IPSS et de qualité de vie avec délai rapide après l'injection de TB-A. MARIA [12] a montré une diminution de 54% et de 65% du score IPSS, respectivement à 1 et 2 mois chez des patients avec un score IPSS initial jugé comme sévère. GUERCINI [13] montre une diminution du score IPSS de 46%, 55%, et 63% respectivement à 1, 2 et 6 mois. CHUANG [14] a mis en évidence une amélioration du score IPSS et de la qualité de vie de 52% et 44% respectivement à 1 mois. Leurs résultats étaient maintenus sur une période d'au moins 6 mois chez des patients en échec du traitement médical par alpha bloquant depuis plus d'un an. La plupart des études ont mis en évidence une diminution significative du volume prostatique, allant de 13 à 68% selon les auteurs. Seules deux études ne mettaient pas en évidence de diminution du volume prostatique après injection de TB-A. CHUANG et al. [14] retrouvaient ainsi une efficacité sur la symptomatologie urinaire sans diminution de volume chez 30% des patients [14].

Dans le cadre de l'évaluation de l'action de la TB-A sur le débit urinaire, là aussi toutes les études ont montré une efficacité significative des injections de TBA sur le débit urinaire maximal (Qmax), confirmant les résultas obtenus sur le score IPSS et de qualité de vie, avec des résultats variables en fonction de la dose injectée, du site d'action et de l'association ou non avec un alpha bloquant. Force est d'admettre que l'on observe un véritable succès chez des patients en impasse thérapeutique. Selon notre analyse, l'augmentation du Qmax constaté était de 15 à 120% et était associée à une diminution du résidu post-mictionnel (RPM) de 34 à 86%. La majorité des études retrouvaient une diminution significative du PSA après injection intraprostatique de la TB-A. L'explication la plus logique de cette baisse était la diminution du volume prostatique. Néanmoins, SILVA et al. [15] ont publié des résultats contradictoires et n'ont pas mis en évidence de diminution du PSA (6 vs 5) malgré une diminution significative du volume de la prostate (70 vs 47). KUO et al. ont étudié l'efficacité de la TB-A chez dix patients en échec de sevrage de sonde urinaire après un traitement médical bien conduit [16]. Une reprise mictionnelle était obtenue chez 80% des patients (Qmax 9,9 ml/s et RPM à 53,9 ml) avec un recul de neuf mois.

## Conclusion

La TB aurait une action multi nodale sur le tissu prostatique en agissant à la fois sur la composante statique et dynamique. Ainsi, l'injection intra prostatique de TB ouvre une nouvelle approche thérapeutique séduisante en s'adressant aux conséquences de tous les types d'adénomes depuis le stade initial de la gêne fonctionnelle à celui de la complication. Les avantages attendus pour le patient sont principalement la diminution des effets secondaires liés aux traitements médicaux et la diminution de la morbidité liée aux traitements chirurgicaux. Des études expérimentales sont nécessaires pour répondre à d'autres questions comme l'effet de ces injections sur d'éventuelles lésions néoplasiques et pour permettre une plus grande connaissance des mécanismes d'action de la toxine botulique sur l'adénome prostatique.

### Etat des connaissances actuelles sur le sujet

- Il existe un véritable enjeu thérapeutique pour les patients trop jeunes ou trop fragiles pour accepter les effets secondaires ou la morbi-mortalité de la chirurgie. Ceci a apporté le développement des techniques mini-invasives, en particulier le traitement par radiofréquence ou par laser. Cependant, leur coût et leur investissement élevés sont des freins important à leur diffusion;
- La TB pourrait se révéler être une alternative judicieuse car facile à mettre en œuvre, sans investissement spécifique et d'un coût raisonnable. Aussi, la TB serait intéressant comme alternative aux traitements médicaux avec une efficacité supérieure, en agissant à la fois sur la composante statique et dynamique de l'HBP, avec un délai d'action plus précoce, et sans effets secondaires. La TB pourrait être une alternative à la chirurgie pour les patients résistants aux traitements médicamenteux. En effet, toutes les études ont mis en évidence une amélioration significative du score IPSS et de qualité de vie avec délai rapide après l'injection de TB.

### Contribution de notre étude à la connaissance

- Cette revue de la littérature présente les premiers résultats de l'injection intra-prostatique de TB soutenant son usage dans l'hyperplasie bénigne de prostate symptomatique.
